# Mate choice for neutral and MHC genetic characteristics in Alpine marmots: different targets in different contexts?

**DOI:** 10.1002/ece3.2189

**Published:** 2016-05-25

**Authors:** Mariona Ferrandiz‐Rovira, Dominique Allainé, Marie‐Pierre Callait‐Cardinal, Aurélie Cohas

**Affiliations:** ^1^ Laboratoire Biométrie et Biologie EvolutiveUniversité de LyonCNRSUMR5558Université Lyon 1F‐69622VilleurbanneF‐69000 LyonFrance; ^2^ Université of LyonVetAgro Sup Campus VetF‐69280Marcy‐L'ÉtoileFrance; ^3^ CREAFCerdanyola del Vallès 08193CataloniaSpain; ^4^ Univ Autònoma de BarcelonaCerdanyola del Vallès 08193CataloniaSpain

**Keywords:** Extra‐pair paternity, inbreeding avoidance, major histocompatibility complex, *Marmota marmota*, mate choice, sexual selection

## Abstract

Sexual selection through female mate choice for genetic characteristics has been suggested to be an important evolutionary force maintaining genetic variation in animal populations. However, the genetic targets of female mate choice are not clearly identified and whether female mate choice is based on neutral genetic characteristics or on particular functional loci remains an open question. Here, we investigated the genetic targets of female mate choice in Alpine marmots (*Marmota marmota*), a socially monogamous mammal where extra‐pair paternity (EPP) occurs. We used 16 microsatellites to describe neutral genetic characteristics and two MHC loci belonging to MHC class I and II as functional genetic characteristics. Our results reveal that (1) neutral and MHC genetic characteristics convey different information in this species, (2) social pairs show a higher MHC class II dissimilarity than expected under random mate choice, and (3) the occurrence of EPP increases when social pairs present a high neutral genetic similarity or dissimilarity but also when they present low MHC class II dissimilarity. Thus, female mate choice is based on both neutral and MHC genetic characteristics, and the genetic characteristics targeted seem to be context dependent (i.e., the genes involved in social mate choice and genetic mate choice differ). We emphasize the need for empirical studies of mate choice in the wild using both neutral and MHC genetic characteristics because whether neutral and functional genetic characteristics convey similar information is not universal.

## Introduction

Sexual selection through female mate choice for genetic characteristics has been suggested to be an important evolutionary force maintaining or regulating genetic variation in animal populations (Jennions 1997; Tregenza and Wedell 2000; Mays and Hill 2004). Males are then supposed to be chosen based on genetic characteristics that contribute to enhance the quality of the offspring produced (Kokko et al. 2003; Mead and Arnold 2004). Three nonexclusive preferences of females for the males' genetic characteristics have been proposed (Mays and Hill 2004; Neff and Pitcher 2005; Roberts et al. 2006): a preference for males possessing specific alleles (i.e., good genes *sensu stricto*), for heterozygous males (i.e., good genes as heterozygosity) and for compatible males. If specific alleles are the target of choice, all females are expected to preferentially mate with males possessing specific alleles that confer a fitness advantage to their offspring (Mays and Hill 2004). If heterozygosity is the target of choice, all females are expected to mate with heterozygous males since heterozygous males should be more fit and therefore should not only provide more direct benefits to the females and their offspring than homozygous males, but also heterozygous males may be more likely to produce heterozygous offspring (Mays and Hill 2004; Roberts et al. 2006)). Finally, if genetic compatibility is the target of choice, females are expected to choose mates based on a relative criterion specific to each female (Trivers 1972; Zeh and Zeh 1996, 1997; Neff and Pitcher 2005), and females should mate with unrelated or genetically dissimilar males to produce heterozygous offspring with higher fitness (Mays and Hill 2004).

However, despite more than two decades of interest, the genetic targets of female mate choice are not clearly identified. Indeed, it is still unclear whether female mate choice for genetic characteristics is based on genome‐wide genetic characteristics, on particular functional loci or on both since there is a limited number of studies considering both types of genetic targets (Table 1). Moreover, the genetic targets of female mate choice could be different from one species to the others. Indeed, population genetic structure, dispersal patterns, social constraints, or parasite pressures, which vary between species, may determine which genetic characteristics are targeted by female mate choice.

**Table 1:** Summary of empirical studies carried out in the wild examining female mate choice using both neutral and MHC genetic markers

Scientific name (reference)	Type of choice	Microsatellites		MHC markers			Correlation between genetic markers
		Good genes as heterozygosity	Compatibility	Good genes sensu stricto	Good genes as heterozygosity	Compatibility	
*Sphenodon punctatus* (Miller et al. 2009)	Social versus random	na	±	na	na	±	na
	Mated versus nonmated	=	na	na	=	na	
*Acrocephalus sechellensis* (Richardson et al. 2005)	Social versus random	na	na	na	=	=	Yes
	Occurrence of EPP	na	=	na	+	=	
	Social versus extra‐pair	na	na	na	+	=	
*Geothlypis trichas* (Bollmer et al. 2012)	Social versus random	na	=	=	±*	=	na
	Occurrence of EPP	na	=	=	=	±§	
	Social versus extra‐pair	na	=	=	=	=	
*Passer domesticus* (Bichet et al. 2014)	Social versus random	na	=	na	na	=	na
	Extra‐pair versus random	na	=	na	na	=	
	Occurrence of EPP	na	=	na	na	na	
	Social versus extra‐pair	na	+	na	na	+	
*Carpodacus erythrinus* (Winternitz et al. 2015)	Social versus random	=	=	na	=	=	no
	Genetic versus random	+	=	na	=	=	
	Occurrence of EPP	+	=	na	=	=	
	Social versus extra‐pair	=	=	na	=	=	
*Microcebus murinus* (Schwensow et al. 2008b; Huchard et al. 2013)	Social versus random	+	=	=	+	+	no
	Social versus random	na	+	na	=	±#	Yes
*Cheirogaleus medius* (Schwensow et al. 2008a)	Social versus random	+	=	+	+	+	No
	Occurrence of EPP	=	=	=	=	+	
	Genetic versus random	+	=	=	=	=	
*Mandrillus sphinx* (Setchell et al. 2010)	Reproductor versus random	±	±	na	+	+	Yes

na: not applicable or not studied; +: Evidence of a female mate choice for the studied genetic marker; =: No evidence of a female mate choice for the studied genetic marker; ±: Evidence of a female mate choice mixed; *: Evidence of a female mate choice for MHC class I alleles and no evidence for MHC class II alleles; §: Evidence of a female mate choice for MHC class II alleles and no evidence for MHC class I alleles; #: Evidence of a female mate choice for MHC DRB locus and no evidence for MHC DQB locus.

Mate choice based on genome‐wide genetic characteristics can occur when female preferences translate into the production of genome‐wide heterozygous offspring (i.e., indirect benefits), because more heterozygous offspring often present a higher fitness (Crnokrak and Roff 1999; Keller and Waller 2002; Coltman and Slate 2003; Oh and Badyaev 2006; but see Kokko and Ots 2006). Females are then expected to avoid mating with homozygous (good genes as heterozygosity hypothesis). Nevertheless, mating with heterozygous males is more likely to confer direct than indirect benefits (Brown 1997; Mays and Hill 2004). Females are also expected to avoid mating with related partners (genetic compatibility hypothesis), especially in those species where strong inbreeding depression occurs (Tregenza and Wedell 2000). Although a choice for dissimilar males (i.e., genetic compatibility hypothesis) could be advantageous for female fitness, this advantage could be, in certain cases, counterbalanced by outbreeding depression (Bateson 1983; Thornhill 1993) and a choice for intermediately dissimilar males could be favored (Penn and Potts 1999).

If female mate choice is based on characteristics at particular functional loci, the major histocompatibility complex (MHC) appears as a likely target. Indeed, the MHC is a multigene family present in all jawed vertebrates (Kelley et al. 2005) playing a critical role in vertebrate disease resistance by initiating immune response (Hedrick 1994). Specifically, transcript molecules from MHC class I and II genes typically recognize intracellular and extracellular pathogens, respectively. Females may choose their mates to produce offspring possessing MHC alleles conferring resistance to pathogens (Takahata and Nei 1990). Associations between the presence of a specific MHC‐allele and the resistance to a pathogen have been highlighted (Harf and Sommer 2005; Kloch et al. 2010; Oppelt et al. 2010; Schwensow et al. 2010; Cutrera et al. 2011). Females are then expected to prefer males possessing these specific MHC alleles (good genes hypothesis sensu stricto).

Also, given that MHC genes are co‐dominantly expressed, MHC‐heterozygous individuals are expected to have a higher probability of recognizing diverse pathogens as they are expected to increase the diversity of antigens presented to T cells than less diverse individuals (Doherty and Zinkernagel 1975; Hughes and Nei 1989; Penn et al. 2002). Accordingly, a negative association between MHC heterozygosity and parasite load has been reported in different species (Penn et al. 2002; Froeschke and Sommer 2005; Lenz et al. 2009; reviewed in Sin et al. 2014). Thus, females are expected to prefer MHC‐heterozygous males (good genes as heterozygosity hypothesis), as MHC‐heterozygous males are expected to be less infected by parasites as well as to transmit lower parasite loads toward their offspring (i.e., direct benefits). Moreover, mating with MHC‐heterozygous males may result in MHC‐heterozygous offspring.

Alternatively, females may choose their mates to produce heterozygous offspring at MHC genes by mating with MHC‐dissimilar males (genetic compatibility hypothesis). High heterozygosity at the MHC could be advantageous but could also promote negative T‐cell selection (i.e., a process removing T cells that bind too strongly to self peptides) (Starr et al. 2003), leading to a reduction in the diversity of T‐cell receptors (Lawlor et al. 1990; Nowak et al. 1992) and, in turn, to a reduction in the resistance to pathogens. To avoid such deleterious effects, a female choice for intermediately MHC‐dissimilar males might be favored. However, female preference for MHC dissimilarity may also occur to avoid mating with related partners (Potts and Wakeland 1990; Penn and Potts 1999). Indeed, MHC loci being among the most polymorphic loci in vertebrates, individuals sharing MHC alleles are expected to be related (Bernatchez and Landry 2003). However, this assumption seems not to hold in all cases (Table 1).

Here, we investigated, using microsatellite and MHC markers, whether females base their mate choice on neutral and/or on particular functional loci in the Alpine marmot (*Marmota marmota*). Mate acquisition in Alpine marmots is a competitive process because reproduction is mostly monopolized by dominant individuals within the family group, although extra‐pair paternities (EPP) occur (Cohas et al. 2008). Whereas social mate choice might be constrained by the necessity to acquire a dominant position before reproduction, extra‐pair mate choice may be a way for female Alpine marmots to adjust their mate choice and to copulate with males possessing peculiar genetic characteristics. We first investigated whether neutral and functional loci convey similar information by testing whether microsatellites and MHC loci characteristics were correlated. Secondly, we tested whether social mate choice and the occurrence of EPP depended on neutral (microsatellites) and/or MHC characteristics. For that, we first investigated whether the presence of specific MHC alleles of social males, the genetic diversity of social males and the genetic compatibility of social pairs differed from those expected under random mate choice (both diversity and compatibility measured at microsatellites and at MHC loci). Secondly, we investigated whether the presence of specific MHC alleles of social males, the genetic diversity of social males and the genetic compatibility of social pairs influenced the occurrence of EPP.

## Materials and Methods

### Studied species

The Alpine marmot is a hibernating ground‐dwelling squirrel, living in family groups from two to 20 individuals, composed of a dominant pair, sexually mature (2 years and older) and immature (yearlings) subordinates of both sexes, and pups (Allainé 2000). Within family groups, reproduction is mainly monopolized (see below) by the dominant pair and the dominant female gives birth to a litter of 1–7 pups (median = 4) once a year. Dominants generally inhibit reproduction of same‐sex subordinates through aggressive behavior (Arnold and Dittami 1997; Hackländer et al. 2003), resulting in a high level of corticosteroids that limits testes maturation and spermatogenesis in subordinate males (Arnold and Dittami 1997), and in the failure of embryo implantation and development in subordinate females (Hackländer et al. 2003). If dominant females successfully monopolize all reproduction, dominant males do not always reach full monopolization of reproduction and EPP occurs (Cohas et al. 2008). Lardy et al. (2012) suggested that the control of many subordinates is energetically costly for dominant males leading them to lose some reproduction and/or dominance. As a result, EPP increases with the number of sexually mature subordinate males present in the family group (Cohas et al. 2006; Lardy et al. 2012). Sexually mature subordinates of both sexes can reach dominance by replacing their fathers or mothers. Otherwise, mature subordinates disperse to reach dominance mainly by replacing a dominant from another territory or rarely by establishing a new territory (only five cases observed between 1990 and 2015 in the studied population). During dispersal, males can gain EPP (Cohas et al. 2006).

### Field methods and sample collection

Data were collected from 1990 to 2010 in a wild population of Alpine marmots located in the Nature Reserve of La Grande Sassière (at 2340 m a.s.l., French Alps, 45°29′N, 65°90′E). Marmots belonging to 25 territories have been captured every year from mid‐April to mid‐July using two‐door live traps baited with dandelions (*Taraxacum officinale*) placed near the entrances of the main burrows in order to assign each captured individual to its family group. Traps were checked every half an hour to limit the time a marmot spent in a trap and thus exposure to unfavorable weather conditions. Once trapped, marmots were placed in an opaque bag and brought to a cabin where handling occurred. They were placed in a calm and cool room for 5 min to recover from the stress of transport and were then tranquillized. Individuals were anaesthetised with Zolétil 100 (0.1 mL kg^−1^), sexed, aged from their size (up to 3 years), and their social status was determined according to scrotal development for males and teat development for females. All individuals were marked using a transponder (model ID100, 0.9 cm long, <0.1 cm in diameter, Trovan Ltd., http://www.trovan.com/) injected under the skin of the neck. The implantation of the transponder has no obvious adverse effects and no migration of the chip from the implantation site or infection has been observed. A numbered metal ear tag (1 cm × 3 mm) was placed on the right ear of females and on the left ear of males. An additional colored plastic ear tag (<1 cm^2^) was placed on the opposite ear of dominant individuals. For genetic analyses, we collected hair from all individuals captured since 1992, and tissue biopsies from the flank of individuals since 1997 which consist in removing a piece of skin (<1 mm^3^) with a biopsy punch (Alcyon, Lyon, France). The marking and biopsies did not cause any bleeding. The implantation of the chip under the skin of the neck and the tissue removal by biopsy were superficial and did not require the use of analgesic. Handling lasted a maximum of 10 min. The recovery did not require the use of an antidote. To recover, marmots were placed again in a calm and cool room for 15 min until they were able to walk. All tranquillized marmots recovered well and no adverse effects have been noticed: all individuals were observed alive the day after their capture. Tranquillizing pregnant or lactating females did not have any obvious impact on offspring as all the females successfully raised offspring to weaning. Overall, individuals were absent from their territory for a maximum of 40 min. We never observed exclusion from the territory for any individual of any age following capture.

The composition of family groups was assessed from both capture–recapture data and intensive observations (see Cohas et al. 2008 for details on observation protocol). The number of subordinates of each sex and age class (pup, yearling, 2 years old, and adult) was assessed for each family group and scent marking behavior was used to confirm the identity of the dominant pair (Bel et al. 1999). Moreover, the date of pup emergence from their natal burrows and the litter size were assessed through daily observations. Virtually all emerged offspring were trapped either with smaller two‐door live traps or by hand within 3 days of emergence.

The field work conducted complies with French laws. All the handling and sampling were done by A.C and M.F.R. who are authorized for experimentation with animals by the French Ministry of Agriculture and Fisheries (diploma nos 0ETRY20090520 and R45GRETAF110). The protocol was approved by the ethical committee of the University Claude Bernard Lyon 1 no. BH2012‐92 V1.

### Microsatellite genotyping

A total of 1045 individuals were genotyped at sixteen microsatellites: SS‐Bibl1, SS‐Bibl18, SS‐Bibl20, SS‐Bibl31, SS‐Bibl4 (Klinkicht 1993); MS41, MS45, MS47, MS53, MS56, MS6, ST10 (Hanslik and Kruckenhauser 2000); Ma002, Ma018, Ma066, Ma091 (Da Silva et al. 2003) (see Appendix S1 for details on genotyping methods and microsatellite characteristics).

### MHC genotyping

A total of 1025 individuals were genotyped at two MHC loci leading to polymorphic proteins – one locus from MHC class I exon 2 (*Mama‐UD;* three alleles resulting in two different proteins) and one locus from MHC class II DRB loci (*Mama‐DRB1*; eight alleles resulting in seven different proteins) (Kuduk et al. 2012) – using three genotyping methods: next generation sequencing, Sanger sequencing and inference based on mother‐father‐offspring triads (Ferrandiz‐Rovira et al. 2015; see Appendix S1 for details on the three genotyping methods and MHC characteristics).

### Parentage analysis

Based on the 16 microsatellites, parentage analyses were performed on 521 pups in two ways. First, the genotypes of each pup and of the dominant female were compared to check maternity. No mismatch between the putative mother and one of its pups was found. We then defined a pup as a within‐pair young (WPY) if no mismatch was observed with the dominant male genotype and as an extra‐pair young (EPY) if at least one mismatch was observed with the dominant male genotype (one to nine mismatches observed). For 14 pups, exclusions of paternity were based on only one mismatch with the dominant male. We considered unlikely that pups with one mismatch were fathered by the dominant male, because (1) genotyping error rate was low (0.0003, for details see Cohas et al. 2008), (2) all these offspring and their parents were retyped and their genotypes confirmed, (3) the average mutation rate for microsatellites is low (1.67 × 10^−4^ per generation in *M. marmota*, Rassmann et al. 1994), and (4) no mismatch with the putative mother has been found. The genotypes of the pups not fathered by the dominant male were then compared to those of all sexually mature males present in their family as well as to sexually mature males born in the remaining studied territories. Finally, remaining pups were considered as fathered by unknown males likely originating from the neighborhood of the studied area and dispersing through it.

Second, parentage analysis was repeated using the software CERVUS 3.0.3 (Kalinowski et al. 2007) with the following settings: 20 candidate fathers per offspring, 98% of candidate parents sampled, error rate of 1% to allow for mistyping and for mutations or null alleles, and assignment at the 95% strict confidence level. We then ran the parentage analyses with the mother identity known and all the candidate fathers. We considered as candidate fathers the sexually mature males present (observed or captured at the year *t*) and potentially present (observed or captured at year *t−1* and/or *t + 1*) in the year *t*. The average nonexclusion probability for one candidate father given the genotype of the known mother was inferior to 0.002. The parentage relationships of the pups established by genetic exclusion were confirmed by parentage analyses except for four pups where a subordinate of the family group was more likely to be the father than the dominant male. The comparison between the MHC genotypes obtained for dominant and subordinate males confirmed that three of these four pups were fathered by the dominant male. The remaining pup could still be assigned to both the dominant and a subordinate male when considering these additional markers. However, the sexual organs of the putative subordinate father showed no sign of development at capture. We consequently parsimoniously considered this pup to be fathered by the dominant male.

### Genetic characteristics estimators

To investigate the three nonexclusive preferences of females – preference for males possessing specific alleles (i.e., good genes sensu stricto), for heterozygous males, (i.e., good genes as heterozygosity) and for compatible males – we estimated whether (1) males possess specific MHC alleles, (2) the genetic diversity of males and (3) the genetic compatibility between males and females both at microsatellites and MHC loci (Table 2).

**Table 2:** Summary of the estimators and results obtained concerning females' preferences for genetic characteristics in the Alpine marmot (*Marmota marmota*)

Genetic markers	Hypothesis	Estimators	Are social males and social pairs genetically different than expected from random mate choice?	Does EPP depend on genetic characteristics of social males and social pairs?	
				Presence of EPY	Number of EPY
Microsatellites	Good genes as heterozygosity	Standardized heterozygosity (SH)	No	No	No
	Compatibility	Relatedness (*R*_qg_)	No	**Yes** High presence and number of EPY when social pairs have low and high genetic similarity	
MHC class I	Good genes sensu stricto	Mama‐UD*02	No	No	
	Good genes as heterozygosity	MHC class I protein diversity	No	No	
	Compatibility	MHC class I protein dissimilarity	No	No	
MHC class II	Good genes sensu stricto	Mama‐DRB1*01, *02,*03,*06,*07,*08	No	No	
	Good genes as heterozygosity	MHC class II protein diversity	No	No	
	Compatibility	MHC class II protein dissimilarity	**Yes** Social pairs have higher dissimilarity than candidate pairs	Trend High occurrence of EPP when social pairs have low genetic dissimilarity	**Yes** High number of EPY when social pairs have low genetic dissimilarity

*P* < 0.05 in the statistical tests are indicated in bold.

To test whether female mate choice is based on genome‐wide genetic characteristics, the neutral genetic diversity of each male and the neutral genetic compatibility of each pair of individuals were estimated, over the 16 microsatellite loci. Whether a moderate number of microsatellites are sufficient to estimate individual genome‐wide characteristics has been challenged in multiple occasions (e.g., Miller et al. 2014). However, in our population, both the pedigree‐based and the Queller & Goodnight's relatedness are strongly correlated (see more details on Appendix S2), suggesting that genetic characteristics calculated at our panel of 16 microsatellites should be representative of genome‐wide patterns.

The neutral genetic diversity was measured by the standardized heterozygosity (SH, Coltman et al. 1999) calculated with the R function “GENHET v3.1” (Coulon 2010). Additionally, the neutral genetic diversity was estimated by the internal relatedness (Amos et al. 2001) and by the homozygosity by locus (Aparicio et al. 2006). The neutral genetic compatibility was estimated by Queller & Goodnight's relatedness (*R*_qg_, Queller and Goodnight 1989) using custom made scripts written in R software version 3.0.3 (R Development Core Team 2014). The neutral genetic compatibility was further estimated by the Lynch & Ritland's estimator (Lynch and Ritland 1999) and Identity (Belkhir et al. 2002). As the three neutral genetic diversity estimators (all *ρ *> 0.94) and the three neutral genetic compatibility estimators (all *ρ *> 0.78) were highly correlated and the results found were independent of the estimator used, we only present the results using the standardized heterozygosity and the Queller & Goodnight's relatedness.

To test whether female mate choice is based on genetic characteristics at particular functional loci, the presence of specific MHC proteins and the diversity of the MHC proteins produced by each male, as well as the compatibility of the MHC proteins produced by each pairs, were estimated over the two MHC loci, *Mama‐UD* and *Mama‐DRB1,* separately because MHC class I and II genes are under different selective pressures in the Alpine marmot (Kuduk et al. 2012), suggesting that both classes could convey different information. To avoid statistical problems, we have considered only proteins produced by more than 5% and less than 95% of individuals (Table S5 in Appendix S1) when estimating the presence of specific MHC proteins on female mate choice. Then, for each retained MHC protein, we noted its presence (1) or its absence (0) in individuals. The MHC genetic diversity was estimated as the MHC protein diversity calculated as the number of different functional MHC proteins a male produces. The MHC genetic compatibility was estimated as the MHC protein dissimilarity calculated as the number of functional proteins a male produces that differed from the ones produced by the female. For the MHC protein diversity and the MHC protein dissimilarity, the protein translated by the allele Mama‐DRB1*08 was considered non‐functional as Mama‐DRB1*08 has a stop codon (Kuduk et al. 2012). However, as the presence of the allele Mama‐DRB1*08 could have a deleterious effect, the presence of this specific allele was kept under consideration.

### Statistical analyses

#### Are genetic characteristics correlated?

To test whether neutral and MHC estimators convey similar information, Spearman's rank correlation was calculated between standardized heterozygosity and MHC protein diversity of the dominant males. Similarly, Spearman's rank correlation was calculated between Queller & Goodnight's relatedness and MHC protein dissimilarity of the social pairs.

#### Are social males and social pairs genetically more different than expected from random mate choice?

If there is a choice for genetic characteristics, one should expect genetic characteristics to differ between observed and candidate mates. Hence, for each reproductive event of each female (from 337 to 356 reproductive events depending on the genetic estimators considered), the genetic characteristics of observed social males (from 97 to 104 males) and of observed social pairs (from 146 to 158 pairs) were compared to the genetic characteristics of candidate males and pairs using bootstrap tests. A male was included in the pool of candidate mates for a given reproductive event of a given female if he met two criteria: (1) he was present in the population (i.e., he was observed and/or captured a given year); (2) he was at least 3 years old (2‐year‐old males rarely reach dominance: 17 of 116 males and the results including males of at least 2 years old were qualitatively similar, we thus only present the results including males of at least 3 years old). Given that only the dominant female reproduces, only one female is considered for each family. The distributions of the differences between genetic estimators of observed versus candidate males (and pairs respectively) were generated under the null hypothesis of random mate choice by randomly allocating, for each reproductive event of each female, a given male as her social male and one of the other males as the candidate male. To take into account the observed distribution of dispersal distances (see Appendix S3 for more details on males' dispersal distances), the probability to allocate a given male was weighted according to its distance with the focal female. This bootstrap procedure was repeated 1000 times using the R package “boot” (Canty and Ripley 2014). In order to test whether genetic characteristics estimators of social males (and of social pairs, respectively) differed from those expected under the hypotheses of random mate choice, the mean observed difference between genetic estimators of the truly observed social males (and pairs) (observed mean) and the candidate males (and pairs) were compared to the mean simulated difference obtained from the random distributions. The exact two‐tailed *P* was computed as the proportion of simulations displaying a greater mean difference than the observed mean difference plus the proportion of simulations displaying a lower mean difference than the symmetrical (relative to the simulated mean difference) of the observed mean difference.

#### Does EPP depend on genetic characteristics of social males and of social pairs?

To determine whether the EPP depended on the social males' and social pairs' genetic characteristics, the presence and the number of EPY within a litter were established for the 145 litters (produced by 83 different social pairs, 39 being present between 2 and 5 years), for which the parentage relationships were established for all pups and the number of mature male subordinates present in the family group was known.

Generalized estimating equations (GEEs) (Liang and Zeger 1986) were used to account for repeated measures of social pairs. GEEs were chosen as they make broader hypotheses about data structure and are better adapted to departure from normality of random effects and small sample sizes within clusters than generalized mixed models (Carlin et al. 2001; Zuur et al. 2009). An exchangeable correlation matrix was chosen to specify the same correlation between all observations of a given pair (this is analogous to the correlation structure derived from specifying the identity of the pair as a random factor in a mixed model, Horton and Lipsitz 1999).

Two series of GEE models were constructed using the R package “geepack” (Halekoh et al. 2006) with the presence of EPP and the number of EPY within litters as the dependent variables, respectively. In the models with the presence of EPP, GEEs were used with a logit link and a variance given by a binomial distribution whereas a logarithm link and a variance given by a Poisson distribution was used when modeling the number of EPY. In all models, litter size and the number of sexually mature male subordinates present in a given family were included as fixed effects to control for a potential confounding effect of litter size on the number of EPY in a litter and to control for the fact that EPP increases with the number of sexually mature males in Alpine marmots (Cohas et al. 2006; Lardy et al. 2012). Queller & Goodnight's relatedness between social partners was further included as a fixed effect in all models since it affects the presence of EPY in Alpine marmots (Cohas et al. 2008). For each model, genetic estimators were then added separately. A linear effect was considered for all genetic diversity estimators while both a linear effect and a quadratic effect were considered for all genetic compatibility estimators to test for a choice for intermediate dissimilarity. As genetic estimators were not available for all males and pairs, sample sizes varied from 134 to 140 litters in the statistical analyses.

## Results

### EPP pattern

A total of 521 pups from 145 different litters were retained from families where the number of sexually mature male subordinates (mean ± SD = 0.83 ± 1.23; range: 0–6) and all pups of the year were known. The mean number of pups within a litter was 3.59 ± 1.23 (±SD) (range: 1–7). From a total of 521 pups, 38 (7.29%) were found to be EPY and from the 145 examined litters, 20 (13.79%) contained at least one EPY. From these 20 litters, 9 (45%), 6 (30%), 4 (20%) and 1 (5%) contained one, two, three, and five EPY, respectively. Four of these 20 litters were sired only by the extra‐pair male (EPM) whereas the remaining 16 were mixed litters (pups sired by both the social and an extra‐pair male). The mean proportion of EPY within the mixed litters was 0.42 ± 0.16 (±SD) (range: 0.20–0.75). From the 20 litters containing at least one pup fathered by an EPM, the EPM was a subordinate of the family in 9 (45%) litters, a dispersing individual born in the study population in 3 (15%) litters and by an unknown male in the remaining 8 litters (40%).

### Are genetic characteristics correlated?

Standardized heterozygosity (neutral diversity) was not correlated to MHC protein diversity regardless of the MHC loci considered (MHC class I: *ρ *= 0.03, CI 95% = −0.17, 0.22, *N* = 104, *P* = 0.78; MHC class II: *ρ *= 0.13, CI 95% = −0.07, 0.31, *N* = 105, *P* = 0.20). A slight negative correlation (*ρ *= −0.19; CI 95% = −0.34, −0.03; *n* = 152, *P* *=* 0.02; that is, individuals more similar at microsatellites loci are more similar at MHC class II proteins) was found between Queller & Goodnight's relatedness (neutral dissimilarity) and MHC class II protein dissimilarity but not with MHC class I protein dissimilarity (*ρ *= −0.05, CI 95% = −0.21, 0.11, *N* = 148, *P* *=* 0.56).

### Are social males and social pairs genetically different than expected from random mate choice?

Social males did not produce more specific MHC proteins (Table 3) nor were more genetically diverse (both at neutral and MHC loci, Table 3) than expected under random mate choice, contrary to the predictions of the good genes sensu stricto and of the good genes as heterozygosity hypotheses, respectively.

**Table 3:** Genetic characteristics at the neutral genetic characteristics (microsatellites), the functional MHC class I loci and the functional MHC class II loci of social males and pairs compared to the ones of candidate males. SH: standardized heterozygosity; *R*_qg_: relatedness. *R*_qg_−*R*_qg__intermediate: absolute difference between the observed value of the relatedness and the intermediate value of the relatedness (0.17) obtained from the generalized estimating equation model of the number of EPY within litters. *P *<* *0.05 are indicated in bold

Genetic markers	Hypothesis	Independent variable	Observed mean	Observed difference	Simulated difference [CI 95%]	*P*
Microsatellites	Good genes as heterozygosity	SH	0.95	−0.03	−0.003 [−0.03, 0.02]	0.09
	Compatibility	*R*_qg_	0.10	0.01	0.003 [−0.02, 0.03]	0.09
		*R*_qg__*R*_qg__intermediate	0.20	−0.004	−0.01 [−0.02, 0.01]	0.70
MHC class I	Good genes sensu stricto	Mama‐UD*02	0.15	0.003	0.01 [−0.02, 0.05]	0.71
	Good genes as heterozygosity	MHC class I protein diversity	1.15	0.003	0.01 [−0.03, 0.04]	0.63
	Compatibility	MHC class I protein dissimilarity	0.20	0.01	0.02 [−0.02, 0.05]	0.58
MHC class II	Good genes sensu stricto	Mama‐DRB1*01	0.55	−0.04	−0.01 [−0.06, 0.05]	0.25
		Mama‐DRB1*02	0.39	−.01	−0.03 [−0.09, 0.02]	0.30
		Mama‐DRB1*03	0.35	0.04	−0.01 [−0.05, 0.04]	0.26
		Mama‐DRB1*06	0.21	0.03	0.02 [−0.02, 0.06]	0.25
		Mama‐DRB1*07	0.16	0.04	0.03 [0.001, 0.06]	0.25
		*Mama‐DR*B1*08	0.07	0.02	0.02 [0.001, 0.04]	0.47
	Good genes as heterozygosity	MHC class II protein diversity	1.75	0.01	−0.0004 [−0.05, 0.04]	0.67
	Compatibility	MHC class II protein dissimilarity	**0.31**	**0.18**	**0.05 [**−**0.01, 0.12]**	**0.04**

*P* < 0.05 in the statistical tests are indicated in bold.

In agreement with the compatibility hypothesis, social pairs showed a higher protein dissimilarity at the MHC class II locus than expected under random choice (Table 3). However, social pairs were not less related nor more intermediately related than expected under random mate choice and did not show a higher protein dissimilarity at the MHC class I locus than expected under random choice (Table 3).

### Does EPP depend on genetic characteristics of social males and social pairs?

As expected, the presence and the number of EPY within litters increased with the number of sexually mature male subordinates present in the family group (presence of EPY: estimate ± SE = 0.47 ± 0.14, *P* = 0.001; number of EPY: estimate ± SE = 0.27 ± 0.13, *P* = 0.05, *N* = 140) but did not depend on litter size (presence of EPY: estimate ± SE = 0.08 ± 0.17, *P* = 0.65; number of EPY: estimate ± SE = 0.12 ± 0.14, *P* = 0.37, *N* = 140).

Contrary to the prediction of the good genes sensu stricto and of the good genes as heterozygosity hypotheses*,* neither the presence of EPY nor the number of EPY within litters were linked to the production of specific MHC proteins by the social mate (Table 4) or to its genetic diversity (both at neutral and MHC genetic loci, Table 4).

**Table 4:** Generalized estimating equation models showing the effects of the neutral genetic characteristics (microsatellites), the functional MHC class I loci and the functional MHC class II loci of the social males and pairs on both the presence and the number of EPY. All models include the number of sexually mature male subordinates present in a given family, the litter size, the social pair relatedness (*R*_qg_) and its associated quadratic term. *P *<* *0.05 are indicated in bold

Genetic markers	Hypothesis	Independent variable	Presence of EPY		Number of EPY		*N*
			Estimate ± SE	*P*	Estimate ± SE	*P*	
Microsatellites	Good genes as heterozygosity	SH	1.47±1.49	0.32	1.11±1.09	0.30	140
	Compatibility	*R*_qg_	−1.73±0.95	0.07	−1.29±0.83	0.12	140
		(*R*_qg_)^2^	**8.40±2.74**	**0.002**	**5.30±1.64**	**0.001**	140
MHC class I	Good genes sensu stricto	Mama‐UD*02	0.29±0.93	0.75	0.20±0.82	0.81	139
	Good genes as heterozygosity	MHC class I protein diversity	0.44±0.92	0.63	0.64±0.75	0.39	135
	Compatibility	MHC class I protein dissimilarity	−0.19±0.89	0.83	−0.69±0.85	0.41	135
MHC class II	Good genes sensu stricto	Mama‐DRB1*01	0.90±0.67	0.18	1.07±0.69	0.12	139
		Mama‐DRB1*02	0.68±0.59	0.25	0.33±0.56	0.56	139
		Mama‐DRB1*03	−0.41±0.63	0.51	−0.57±0.62	0.36	139
		Mama‐DRB1*06	−0.59±0.83	0.48	0.23±0.76	0.76	139
		Mama‐DRB1*07	−0.80±1.20	0.50	−0.65±1.07	0.54	139
		Mama‐DRB1*08	−2.23±1.41	0.11	−2.32±1.18	0.05	139
	Good genes as heterozygosity	MHC class II protein diversity	0.65±0.79	0.41	1.02±0.67	0.13	139
	Compatibility	MHC class II protein dissimilarity	−0.86±0.46	0.06	−**1.05±0.46**	**0.02**	138

P < 0.05 in the statistical tests are indicated in bold.

**Figure 1:**
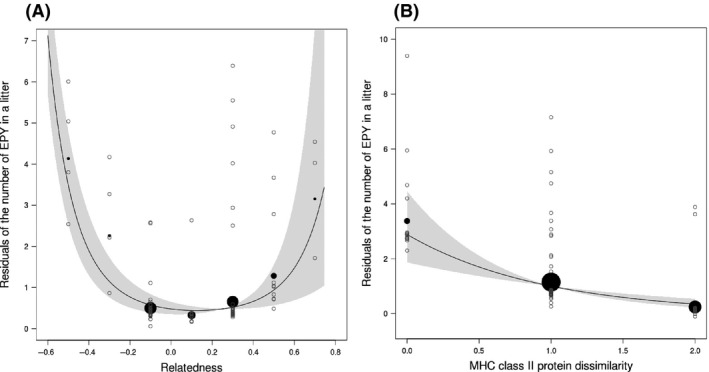
Residual of the number of EPY in a litter as a function of Queller and Goodnight's relatedness between social pairs (A) and as a function of MHC class II protein dissimilarity between social pairs (B). Residuals are corrected for confounding factors (the number of sexually mature male subordinates present in a given family in (A) and for the number of sexually mature male subordinates present in a given family, the social pair Queller and Goodnight's relatedness and its associated quadratic term in (B). Open circles represent the observed residual number of EPY in a litter. Thick lines represent predictions of the model and the gray surface represents standard errors of the fitted model. The black dots represent observed data averaged over classes and their size is proportional to the number of litters within each class (class width 0.2 in A).

In agreement with the compatibility hypothesis, the presence and the number of EPY within litters depended on the relatedness between social pair members. Both the presence and the number of EPY were indeed high when the social males and females were either very genetically similar or dissimilar, as measured by Queller and Goodnight relatedness, but were low when the social pairs showed an intermediate level of neutral genetic similarity (*R*_qg_ of, respectively, 0.14 and 0.17) (Table 4, Fig. 1A). Moreover, the number – and to a lesser extent the presence – of EPY within litters decreased linearly when the social pair MHC class II protein dissimilarity increased (Table 4, Fig. 1B). But, neither the presence nor the number of EPY within litters depended on the MHC class I protein dissimilarity between social pair members.

## Discussion

While a choice based on genome‐wide genetic characteristics has now been extensively studied in wild populations, the existence of a choice based on MHC characteristics has still been seldom studied under natural conditions. Studies considering both types of targets are even scarcer (see Table 1 for a summary of empirical studies carried out in the wild considering both neutral and MHC characteristics) and our study provides evidences that female Alpine marmots base their mate choice on both neutral and MHC characteristics (see Table 2 for a summary of the results). In Alpine marmots, social pairs were not random according to MHC class II protein dissimilarity, and EPP was associated with MHC class II protein dissimilarity between pair members and to neutral genetic similarity (i.e., relatedness). EPPs were rare for high MHC protein dissimilarity and for intermediate relatedness between social pairs' members. These results are in agreement with the genetic compatibility hypothesis rather than with the good genes sensu stricto or with the good genes as heterozygosity hypotheses. Finally, in this species, neutral and MHC characteristics convey different information.

Previous studies based either on neutral characteristics (reviewed in Mays et al. 2008 for birds) or on MHC characteristics (e.g., birds: Ekblom et al. 2004; Westerdahl 2004; Richardson et al. 2005; Bollmer et al. 2012; primates: reviewed in Setchell and Huchard 2010) have found evidence for a choice for good genes sensu stricto, good genes as heterozygosity, compatible genes, all or none. These contrasted empirical results suggest that the species' ecological and biological characteristics might play a significant role on the targeted genes (Setchell and Huchard 2010).

We found no support for the good genes hypothesis sensu stricto. Producing offspring possessing specific MHC alleles is advantageous when specific alleles (often rare alleles) provide a greater resistance against specific parasites than others (Bodmer 1972; Potts and Wakeland 1990; Slade and McCallum 1992). In agreement, many studies have evidenced associations between specific MHC alleles and pathogen resistance (reviewed in Sin et al. 2014). However, in Alpine marmots, mate choice was not based on specific MHC alleles, suggesting that the different MHC proteins considered here may be poorly related to parasite resistance. The good genes as heterozygosity hypothesis was also not supported. Indeed, mate choice was not based on males' heterozygosity both at neutral and MHC loci, suggesting that neither direct nor indirect benefits potentially provided by heterozygous mates are targeted by female Alpine marmots.

Instead, we found support for the genetic compatibility hypothesis. Genetic compatibility was targeted both at neutral and MHC loci. A choice for genome‐wide compatibility is expected in inbred populations where the cost of inbreeding is high (Bernatchez and Landry 2003; Setchell and Huchard 2010; but see Bichet et al. 2014). The cost of inbreeding is likely to be high in the Alpine marmot because juvenile survival decreases with offspring homozygosity (Cohas et al. 2009). Therefore, inbreeding avoidance is expected in the Alpine marmot. However, we did not find that the social pairs were established based on neutral genetic compatibility, suggesting female Alpine marmots do not avoid pairing with related social males. This is because female Alpine marmots likely have reduced opportunity to avoid related social partners. Indeed, more than 48% of individuals from both sexes acquire dominance in their natal territory or in the immediate vicinity resulting in most of the available social males being genetically similar to females (see Appendix S3 for detailed information regarding dispersal patterns). As a result, the Alpine marmot is characterized by a low neutral genetic variability as estimated with allozymes (Preleuthner and Pinsker 1993), minisatellites (Rassmann et al. 1994; Kruckenhauser et al. 1997), and microsatellites (Cohas et al. 2009; but see Goossens et al. 2001) and by a low MHC variability (Kuduk et al. 2012; Ferrandiz‐Rovira et al. 2015).

Choosing an extra‐pair mate with compatible genetic characteristics could be an alternative way for female Alpine marmots to avoid inbreeding. In agreement, a higher occurrence of EPP was found when social pairs have either low or high neutral genetic similarity, suggesting that female Alpine marmots choose to cuckold their social males to gain indirect benefits (e.g., offspring with intermediate outbreeding) and probably resulting in inbreeding avoidance. Unfortunately, we were unable to examine the neutral genetic compatibility of EPM because they were rarely identified. Indeed, from the litters that contained at least one EPY, the genetic father was attributed in 45%, 15%, and 40% of litters to, respectively, a subordinate male of their family, a dispersing individual born in our study population and an unknown male coming from outside of our study population. Thus, no genetic data being available for unknown EPM, our sample is strongly biased toward subordinate males of the family group. However, it is worth to notice that among the subordinate EPM none was a son of the dominant female confirming inbreeding avoidance.

Our results further indicated that females based both the establishment of social pairs and their decision to cuckold their mates on MHC protein dissimilarity, specifically on MHC class II but not on MHC class I. A choice for genetic compatibility at specific loci is expected when heterozygosity at these specific loci confers selective advantage. Parasite pressures could modulate the fitness pay‐offs of MHC‐based mate choice. Females may choose MHC compatible mates to produce MHC‐heterozygous offspring (Takahata and Nei 1990) because the presence of specific alleles conferring resistance to parasite is more likely to occur in heterozygous individuals (Apanius et al. 1997) and because heterozygous offspring are able to recognize a wider range of pathogens (Doherty and Zinkernagel 1975). In accordance, MHC diversity has been found to be negatively associated with parasite load (Penn et al. 2002; Froeschke and Sommer 2005; Lenz et al. 2009). The strong positive selection detected at the antigen binding sites of the MHC class II alleles (MHC class II participates in the immune response toward extracellular parasites) but not of the MHC class I alleles (MHC class I participates in the immune response toward intracellular parasites) of Alpine marmots suggests that high selective pressures are exerted by extracellular parasites (Kuduk et al. 2012). As a matter of fact, while Alpine marmots are usually infested by several parasites – an ectoparasite (Arnold and Lichtenstein 1993) and four intestinal parasites including three helminths species (Bassano et al. 1992; Callait & Gauthier, 2000) – bacteria, and viruses, which are often intracellular parasites, have not been already described in Alpine marmots (Bassano 1996). Similar divergent patterns of mate choice for MHC characteristics according to the MHC class have been already found in blue petrel (*Halobaena caerulea*) (Strandh et al. 2012) and in European badger (*Meles meles*) (Sin et al. 2015). And, the low parasite pressures encountered in insular populations has been hypothesized to be at the origin of the absence of mate choice for MHC class I genes in an insular population of house sparrows (*Passer domesticus*) (Bichet et al. 2014).

Among the correlations calculated between neutral and MHC markers, only one was significant (between relatedness and MHC class II protein dissimilarity) but weak (*R* ^2^ < 4%). We therefore suggest that neutral and MHC characteristics convey different information in the Alpine marmot. This absence of correlation indicates that MHC‐based social pairing and occurrence of EPP were not a way to avoid inbreeding. In species where neutral and MHC characteristics are correlated (Table 1, Gasparini et al. 2015), we cannot exclude the apparent choice for a genetic characteristic to be a by‐product of a choice for the other one (Piertney and Oliver 2006; Kempenaers 2007; Huchard and Pechouskova 2014). For example, in mandrills (*Mandrillus sphinx*), females were found to mate according to both neutral and MHC characteristics (Setchell et al. 2010). However, because neutral and MHC characteristics are correlated in this species, one cannot exclude that evidence for MHC‐dependent female mate choice is a way to avoid inbreeding. Indeed, MHC loci being among the most polymorphic loci in vertebrates, individuals that share MHC alleles are likely to be related (Bernatchez and Landry 2003) and it has been argued that females may avoid inbreeding through MHC‐based female choice (Potts and Wakeland 1990; Penn and Potts 1999). The absence of correlation between neutral and MHC markers (as found in Alpine marmots, see Table 1 for examples of absence of correlation in other species) allows females targeting different genetic characteristics independently in different contexts. In scarlet rosefinches (*Carpodacus erythrinus*), social mate choice does not depend on genetic characteristics (neither at neutral nor at MHC loci), but EPMs are chosen on neutral characteristics but not on MHC characteristics (Winternitz et al. 2015). Also, in fat‐tailed dwarf lemurs (*Cheirogaleus medius*), social mates are primarily chosen according to their heterozygosity (neutral and MHC) and their compatibility with females at MHC loci, while extra‐pair mates are chosen according to their genetic compatibility to the females, but only at MHC loci (Schwensow et al. 2008a). Targeting different genetic characteristics may also result from different constraints on female choice in different contexts. Typically, female Alpine marmots have few opportunities to avoid pairing with an unrelated social partner, but they choose it according to its MHC characteristics. But, female Alpine marmots are less constrained when it comes to choose extra‐pair mates and they may thus seek extra‐pair mates to avoid producing inbred offspring. To better understand the evolutionary causes and consequences of female mate choice, these considerations advocate for the need to not only combine both neutral and MHC characteristics (and not to consider a single target) but also to examine the correlation between them.

## Conclusions

Our results suggest that female Alpine marmots base their mate choice on both neutral and MHC loci. This choice seems to be context‐dependent as indicated by the fact that the genes involved in the establishment of social pairs and in the occurrence of EPP differ. The present study adds to the scarce existing literature considering both neutral and specific loci such as MHC and corroborates the hypothesis that numerous genetic targets of mate choice could occur depending on the considered species or the considered context within a species. Population genetic structure, dispersal patterns, social constraints, or parasite pressures may play a key role in female mating decisions. Whether females actively choose for genetic characteristics or whether this choice is a by‐product of the geographic origins of males remains an open question. In the case of an assessment of genetic characteristics, olfactory cues are likely to be involved. For example, characteristics of chemical compounds have been proven to depend on the genetic characteristics of wild or semi‐free‐ranging individuals (e.g., neutral loci: in ring‐tailed lemurs (*Lemur catta*) (Charpentier et al. 2008) and in black‐legged kittiwakes (*Rissa tridactyla*) (Leclaire et al. 2012); or MHC loci: in mandrills (Setchell et al. 2011)). Exploration of the association between genetic characteristics at either neutral or MHC loci, odor characteristics, and mating preferences in wild populations is a promising avenue of research that awaits further investigation.

## Conflict of Interest

None declared.

## Supporting information


**Appendix S1.** Molecular analyses and genetic markers characteristics.
**Table S1.** Primer pairs used for microsatellite and MHC genotyping.
**Table S2.** Characteristics of 16 microsatellites of Alpine marmots.
**Table S3.** Tags used to barcode individuals for next generation sequencing.
**Table S4.** Allelic frequencies of the two MHC loci of Alpine marmots.
**Table S5**. Number (*N*) and percentage (P) of individuals carrying the 10 MHC polymorphic proteins.

**Appendix S2.** Estimation of relatedness with pedigree and correlations between (1) the relatedness estimated with the pedigree and the relatedness estimated estimated with the Queller and Goodnight (1989) and (2) the relatedness estimated with the pedigree and the MHC proteic dissimilarity.

**Appendix S3.** Effect of the spatial distance on males and pairs genetic characteristics.
