# Supplementary material for: Mate choice for neutral and MHC genetic characteristics in Alpine marmots: different targets in different contexts?
Source: Ecol Evol. 2016 May 25;6(13):4243–57. doi: 10.1002/ece3.2189 (PMC4930977; doi:10.1002/ece3.2189)
Supplement: Supplementary file 1 — Appendix S1. Molecular analyses and genetic markers characteristics. Table S1. Primer pairs used for microsatellite and MHC genotyping. Table S2. Characteristics of 16 microsatellites of Alpine marmots. Table S3. Tags used to barcode individuals for next generation sequencing. Table S4. Allelic frequencies of the two MHC loci of Alpine marmots. Table S5. Number (N) and percentage (P) of individuals carrying the 10 MHC polymorphic proteins. [file ECE3-6-4243-s001.doc]

**Mate choice for neutral and MHC genetic characteristics in Alpine marmots: different targets in different contexts?**

**Appendix S1.** Molecular analyses and genetic markers characteristics

*DNA extraction*

For 1045 individuals, genomic DNA was extracted from 15 to 30 hairs or skin biopsies by incubation at 66°C for 80 min for hairs and at 56°C for 120 min for tissue in 50 μL lysis buffer (20 mM Tris-HCl, 1.5 mM MgCl_2_, 25 mM KCl, 0.5% Tween 20, and 0.1 mg/ml proteinase K), followed by 20 min of proteinase K inactivation at 96ºC.

*Microsatellites genotyping*

Individuals were typed at 16 microsatellite loci: SS-Bibl1, SS-Bibl18, SS-Bibl20, SS-SS-Bibl31, SS-Bibl4 (Klinkicht, 1993); MS41, MS45, MS47, MS53, MS56, MS6, ST10 (Hanslik & Kruckenhauser, 2000); Ma002, Ma018, Ma066, Ma091 (Da Silva *et al.,* 2003) (see Table S1 for primers design). To enable polymerase chain reaction (PCR) multiplexing and subsequent assessment of the allele sizes for all loci, we used primers labeled with FAM, PET, NED and VIC fluorescent dyes (FAM for SS-Bibl1, MS45, Ma066 and Ma091, PET for SS-Bibl18, SS-Bibl20, SS-Bibl4, Ma002 and Ma018, NED for SS-Bibl31, MS47, MS53 and Ma002, and VIC for MS41, MS56, MS6 and ST10). PCR was carried out in three 10 μl reactions (Mix1, Mix2 and Mix3) containing 5 μl of Kit PCR (QIAGEN, Hilden, Germany) and 1 μl of DNA extract with a DNA concentration of 25-100 ng/ml. In addition, Mix1 contained 0.03 μl of each primer for MS45, 0.1 μl of each primer for SS-Bibl31, MS41and ST10, 0.2 μl of each primer for SS-Bibl18 and SS-Bibl4 and 0.3 μl of each primer of Ma002. Mix2 contained 0.05 μl of each primer for MS56 and MS6, 0.1 μl of each primer for MS53 and Ma091, 0.14 μl of each primer for SS-Bibl1, and 0.2 μl of each primer for MS47, Ma018 and Ma066. Mix3 contained 0.2 μl of each primer for SS-Bibl20. Amplifications were carried out in a Mastercycler (Eppendorf, Hambourg, Germany) thermo-cycler with the following cycling conditions: 15 min at 95°C, then 28 cycles for Mix1 and Mix2 and 35 cycles for Mix3 composed of 30 s denaturing at 94°C, 90 s annealing at 57°C, 60 s extension at 72°C, and finally 30 min at 60°C to ensure complete extension. We then added 1.5 μl of Mix1 and 1.5 μl of Mix2 plus 1.5 μl of Mix3 to 0.15 μl of size standard ROX 60-415 and 10 μl of formamide. Electrophoresis was run for 3 h on an automated sequencer ABI 3130 (Applied Biosystems Inc., Foster City, CA, U.S.A.) to determine allele sizes. Microsatellite patterns were examined with Genemapper 4.0 (Applied Biosystems). Depending on the locus, 97-100% of individuals were genotyped and details on microsatellites characteristics are summarized in Table S2.

Hardy-Weinberg equilibrium was tested using “HWE.test” implemented on the library “genetics” (Warnes, 2012) of the R version 3.1.1 (R Development Core Team 2013). These tests were performed on 200 randomized individuals to avoid potential bias caused by family structure and on all cohorts pooled to ensure sufficient sample size. None of the loci showed deviation from Hardy–Weinberg equilibrium after Bonferroni correction (P < 0.05/16 = 0.003), except for Ma002 (χ² = 33.69, P < 0.0001, 10000 replicates).

*MHC genotyping*

Individuals were genotyped at two MHC loci, one from MHC class I exon 2 (*Mama-UD*) and one from MHC class II DRB loci (*Mama-DRB1*). Three different methods were used to genotype individuals: next generation sequencing, Sanger sequencing or were deduced based on mother-father-offspring triads.

*Next generation sequencing*

Two runs of a Roche® 454 FLX sequencing instrument were done. Methods used to genotype individuals at four MHC loci have been described in details elsewhere (Ferrandiz-Rovira *et al.,* 2015) and are briefly summarized here.

Amplification of MHC loci used primers specifically designed to separate each locus and to avoid sequencing several duplicated genes of Alpine marmots (Table S1). A 6-bp tag was added between the adapters and the locus-specific reverse and forward primers to barcode individuals. A minimum of 3-bp differences between tags were used to ensure a very low probability for a read to be assigned to the wrong individual due to a typing mistake in the individual tag (Table S3 for details on tag library). PCR was performed in a Mastercycler (Eppendorf, Hamburg, Germany) in 10 μL of reaction mixture containing 5 μL HotStarTaq Polymerase Master Mix (QIAGEN, Hilden, Germany), 0.2 μL of both primers at 100 μM, 3.6 μL water and 1 μL DNA at a concentration of 30 ng·μL^-1^. The cycling scheme was 95ºC for 15 min, followed by 34 cycles at 95ºC for 30 s, primer-speciﬁc annealing temperature (50ºC for *Mama-UD* and 55ºC for *Mama-DRB1*) and for 30 s, 72ºC for 60 s and a ﬁnal extension step at 72ºC for 10 min. The concentration of the PCR products was measured by fluorometry using the Quant-iT PicoGreen dsDNA Assay Kit (Invitrogen). Equimolar amounts of amplicons were pooled for a given locus and purified using MinElute PCR Purification Kit (QIAGEN) and the four loci were subsequently pooled and sequenced on an eight of a PicoTiterPlate of a Roche® 454 FLX sequencing instrument (576 amplicons/eight).

Although next generation sequencing is a suitable method for large-scale genotyping and generates multiple reads for a given amplicon, it is also prone to errors (Glenn, 2011). Discerning true alleles versus sequencing errors is thus challenging (Babik, 2010). An accurate post-processing of obtained sequences was conducted in four steps in order to ensure the reliability of assigned MHC genotypes: (1) assignment of reads to loci and individuals, elimination of singletons and elimination of reads with inappropriate sizes; (2) elimination of reads with insufficient coverage; (3) determination of alleles; and (4) determination of homozygous and heterozygous amplicons (see Ferrandiz-Rovira *et al.,* 2015 for all details on post-processing). Finally, 1838 genotypes (898 *Mama-UD and* 915 *Mama-DRB1*) were obtained. The reliability of obtained genotypes was assessed through three independent methods: (1) intra-individual next generation sequencing repeatability; (2) comparison of obtained genotypes with Sanger and next generation sequencing; and (3) comparison of the consistency of mother-father-offspring triad genotypes, with an error rate estimated to 0.3% (see Ferrandiz-Rovira *et al.,* 2015 for all details on methods to assess genotype reliability).

*Sanger sequencing*

For Sanger sequencing, PCR amplifications were carried out following the same protocol used for next generation sequencing (with the exception that no individual barcoding or adaptator were added to the primers). 30 μL of PCR products were purified using Axygen® Cleanup Kit for PCR following the manufacturer's instructions. Purified PCR products were single strand sequenced using Dideoxynucleotide Terminator (Dyeterminator, kit BigDye® v.3.1 provided by LifeTechnologies). Sanger sequences are prone to sequencing errors in the 5' region. So, for the polymorphic regions to be on the 3' of the Sanger sequences, we sequenced with the reverse primer, when the polymorphism was at the end of the sequence of interest, and with the forward primer, when the polymorphism was at the beginning of the sequence of interest (MarmR4 (forward) for *Mama-UD* and MM_DRB_R3 (forward) for *Mama-DRB1*). Sequenced products were then purified using AxyPrep Mag Dye Clean (Axygen, following manufacturer’s instructions). The DNA sequencing reactions were then analyzed on an ABI3730XL 96 caps DNA Analyzer (LifeTechnologies) (see Ferrandiz-Rovira *et al.,* 2015 for more details on the protocol). Obtained reads were aligned using the progressive alignment (Feng & Doolittle, 1987) with the default aligning parameters of the CLC Sequence Viewer software free trial version 6.7.1. to assign 120 genotypes (64 *Mama-UD* and 56 *Mama-DRB1*).

*Deduction from parentage relationships*

Thanks to established parentage relationships, homozygous genotypes of both parents at a given MHC loci (obtained either by next generation sequencing or Sanger sequencing) were used to deduce 15 MHC genotypes of their pups (14 at *Mama-UD* and 1 at *Mama-DRB1*). Genotypes were only deduced when, for a given individual, no more genetic material was available or after the failure of next generation sequencing and/or Sanger sequencing. However, we considered as reliable all genotypes inferred using information on parental genotypes since (1) parentage relationships were highly reliable (see methods regarding reliability of parentage analysis) and (2) only 0.3% of MHC genotyping errors were found after an extended validation procedure of obtained genotypes (see Ferrandiz-Rovira *et al.,* 2015 for more details). The bias in the number of deduced genotypes among different loci (14 at *Mama-UD* and 1 at *Mama-DRB1*) is inherent to the high variation in allelic diversity observed among the studied loci (Table S4). For instance, the higher assignation rate of genotypes at *Mama-UD* is a consequence of the existence of only three alleles at this locus, which increases the likelihood of homozygous pairs.

Finally, a total of 1973 genotypes from 1025 individuals were obtained among the four MHC loci (1838 using next generation sequencing, 120 using Sanger sequencing and 15 deduced on mother-father-offspring triads. Details on MHC loci characteristics are summarized in Table S4.

**Table S1** Primer pairs used for microsatellite and MHC genotyping.

| Locus name | Forward primer (sequence 5’ – 3’) | Reverse primer (sequence 5’ – 3’) |
| --- | --- | --- |
| SS-Bibl1 | CTGAAGCAGCCATCCAGTA | TGGTGTTGCCATTGTTCT |
| SS-Bibl18 | ATGGTCATGGAAGGGAAG | GGCATCTTCACAGTTGATC |
| SS-Bibl20 | ATTCTCTAGTCGTTAACAAGAATC | CACCAGTGAAACTACATACAGTG |
| SS-Bibl31 | TTACACCTTCTCTGGCTCC | TCTGAGCGGATTGTCTTTAT |
| SS-Bibl4 | CCTAGGTTCAGTCTTCAACACA | TGGTGTTGCCATTGTTCT |
| Ma002 | CATTTAGACGCACATTTTG | GGGATGGAGAATGAGGAAG |
| Ma018 | ATCCGTCCAATAAAGAAATTC | GTTTCTTGTGGCTCAGTGGTCAGATG |
| Ma066 | AATATGTTAAGGCAGTTCTAGC | GTTTCTTCCTGATATGGAAAGATGATGT |
| Ma091 | CCTGTGTGAGTCCTGGAGTC | AGCCATTTAGGTTACATCTGC |
| MS41 | GGTGTATATGGGAATAGGGGG | GCCTTCAAATCAAAGCAGGTTG |
| MS45 | CTGTCTCTTTGTCCCTGCC | CTCCTTACCATCATCTTTCCG |
| MS47 | CCTGATGTAGTCAGTCAG | TGTGGGAAATGGCACATC |
| MS53 | ATTGAGGAGCAGCATCTAGG | TCAGGGAAAGGCAGACCTG |
| MS56 | CAGACTCCCACCAGTGACC | CCTGATCTATGTAGGTTCCAT |
| MS6 | CTGATGGGGTTAAGATTGCC | CCCCACTGACCCACCTCC |
| ST10 | TTGTGATCCTCCAGGGAGTT | GTGATTTCCAAACCCCATTC |
| *Mama-UD* | MarmMF1 (AyCTCCGTGTCCCGGCCC) | MarmR4 (GCGCAGGGTGTTCAAGCACAT) |
| *Mama-DRB1* | MM_DRB_F1 (GAGTGTCATTTCTCCAACsrGA) | MM_DRB_R3 (TyAmCTCTCCkCTCCACAGTGAA) |

**Table S2** Characteristics of 16 microsatellites of Alpine marmots.

|  | SS-Bibl1 | | SS-Bibl18 | | SS-Bibl20 | | SS-Bibl31 | | SS-Bibl4 | | Ma002 | | Ma018 | | Ma066 | | Ma091 | | MS41 | | MS45 | | MS47 | | MS53 | | MS56 | | MS6 | | ST10 | |
| --- | --- | --- | --- | --- | --- | --- | --- | --- | --- | --- | --- | --- | --- | --- | --- | --- | --- | --- | --- | --- | --- | --- | --- | --- | --- | --- | --- | --- | --- | --- | --- | --- |
|  | Alleles | Freq | Alleles | Freq | Alleles | Freq | Alleles | Freq | Alleles | Freq | Alleles | Freq | Alleles | Freq | Alleles | Freq | Alleles | Freq | Alleles | Freq | Alleles | Freq | Alleles | Freq | Alleles | Freq | Alleles | Freq | Alleles | Freq | Alleles | Freq |
|  | 95 | 0.15 | 132 | <0.01 | 206 | 0.01 | 157 | 0.50 | 175 | 0.13 | 265 | <0.01 | 296 | 0.25 | 231 | 0.63 | 159 | 0.13 | 184 | 0.17 | 107 | 0.40 | 176 | 0.03 | 132 | 0.13 | 104 | 0.02 | 142 | 0.05 | 116 | 0.15 |
|  | 97 | 0.22 | 137 | 0.01 | 208 | 0.19 | 159 | 0.27 | 178 | <0.01 | 271 | 0.19 | 298 | 0.75 | 233 | 0.02 | 167 | 0.09 | 186 | 0.83 | 109 | 0.49 | 180 | 0.26 | 140 | 0.44 | 106 | 0.30 | 158 | 0.88 | 118 | 0.26 |
|  | 101 | 0.44 | 143 | 0.36 | 216 | 0.39 | 161 | 0.18 | 188 | 0.17 | 279 | 0.50 |  |  | 241 | 0.35 | 169 | 0.04 |  |  | 111 | 0.11 | 182 | 0.17 | 142 | 0.42 | 108 | 0.68 | 160 | 0.07 | 120 | 0.22 |
|  | 103 | <0.01 | 145 | 0.13 | 218 | 0.33 | 163 | 0.05 | 190 | 0.66 | 281 | 0.31 |  |  |  |  | 171 | 0.01 |  |  |  |  | 184 | 0.18 | 144 | <0.01 | 110 | <0.01 |  |  | 130 | 0.05 |
|  | 107 | 0.15 | 147 | 0.39 | 220 | 0.08 |  |  | 192 | 0.04 | 283 | <0.01 |  |  |  |  | 173 | 0.17 |  |  |  |  | 186 | 0.33 | 148 | 0.01 |  |  |  |  | 132 | 0.15 |
|  | 109 | 0.04 | 149 | 0.11 | 222 | <0.01 |  |  |  |  |  |  |  |  |  |  | 175 | 0.44 |  |  |  |  | 188 | 0.02 |  |  |  |  |  |  | 134 | 0.14 |
|  |  |  |  |  |  |  |  |  |  |  |  |  |  |  |  |  | 177 | 0.02 |  |  |  |  | 190 | 0.01 |  |  |  |  |  |  | 136 | 0.03 |
|  |  |  |  |  |  |  |  |  |  |  |  |  |  |  |  |  | 179 | 0.09 |  |  |  |  |  |  |  |  |  |  |  |  |  |  |
|  |  |  |  |  |  |  |  |  |  |  |  |  |  |  |  |  | 188 | <0.01 |  |  |  |  |  |  |  |  |  |  |  |  |  |  |
| Nind | 1,045 | | 1,042 | | 1,010 | | 1,043 | | 1,041 | | 1,015 | | 1,025 | | 1,035 | | 1,038 | | 1,032 | | 1,040 | | 1,033 | | 1,038 | | 1,037 | | 1,033 | | 1,031 | |

**Table S3** Tags used to barcode individuals for next generation sequencing. All combinations of a forward and a reverse tags within each of the two groups were used.

| Group | Forward tag | Reverse tag |
| --- | --- | --- |
| 1 | AACCGA | TTGAGT |
|  | AAGTGT | AAGCAG |
|  | AGTGTT | TTGCAA |
|  | CCGCTG | CACGTA |
|  | AACGCG | TAACAT |
|  | GGCTAC | GGTCGA |
|  | TTCTCG | CTTGGT |
|  | TCACTC | TCCAGC |
|  |  | ACTTCA |
|  |  | GCGAGA |
|  |  | TGGAAC |
|  |  | CGAATC |
| 2 | GAACTA | TTGAGT |
|  | CACAGT | AAGCAG |
|  | CAATCG | TTGCAA |
|  | CCGTCC | CACGTA |
|  | AAGACA | TAACAT |
|  | GGTAAG | GGTCGA |
|  | ATAATT |  |
|  | CGTCAC |  |

**Table S4** Allelic frequencies of the two MHC loci of Alpine marmots**.**

|  | *Mama-UD* alleles | *Mama-UD* allelic freq. | *Mama-DRB1* alleles | *Mama-DRB1* allelic freq. |
| --- | --- | --- | --- | --- |
|  | *01 | 0.73 | *01 | 0.39 |
|  | *02 | 0.08 | *02 | 0.23 |
|  | *03 | 0.19 | *03 | 0.13 |
|  |  |  | *04 | 0.02 |
|  |  |  | *05 | 0.03 |
|  |  |  | *06 | 0.07 |
|  |  |  | *07 | 0.09 |
|  |  |  | *08 | 0.04 |
| Nind | 976 | | 972 | |

**Table S5**. Number (N) and percentage (P) of individuals carrying the 10 MHC polymorphic proteins.

| Loci | Allele | N | P |
| --- | --- | --- | --- |
| *Mama-UD* | *Mama-UD**01 and/or *Mama-UD**03**^1^** | 970 | 99 % |
|  | ***Mama-UD**02** | **143** | **15 %** |
| *Mama-DRB1* | ***Mama-DRB1**01** | **618** | **64%** |
|  | ***Mama-DRB1**02** | **409** | **42%** |
|  | ***Mama-DRB1**03** | **229** | **24%** |
|  | *Mama-DRB1**04 | 37 | 4% |
|  | ***Mama-DRB1**05** | **58** | **6%** |
|  | ***Mama-DRB1**06** | **135** | **14%** |
|  | ***Mama-DRB1**07** | **170** | **17%** |
|  | ***Mama-DRB1**08^2^** | **78** | **8%** |

Retained proteins for statistical analysis (percentage of individuals carrying a given protein > 5% and < 95%) are indicated in bold. ^1^: *Mama-UD*01* and *Mama-UD*03* produce the same protein and thus are counted together. ^2^: non-functional protein.

**References**

Babik, W. (2010). Methods for MHC genotyping in non-model vertebrates. *Molecular Ecology Resources*, *10*, 237-251.

Da Silva, A., Luikart, G., Allainé, D., Gautier, P., Taberlet, P., & Pompanon, F. (2003). Isolation and characterization of microsatellites in European alpine marmots (*Marmota marmota*). *Molecular Ecology Notes*, *3*, 189-190.

Ferrandiz-Rovira, M., Bigot, T., Allainé, D., Callait-Cardinal, M-P., & Cohas, A. (2015). Large-scale genotyping of highly polymorphic loci by next generation sequencing: how to overcome the challenges to reliably genotype individuals? *Heredity, 114*, 485-493*.*

Feng, D. F., & Doolittle, R. F. (1987). Progressive sequence alignment as a prerequisite to correct phylogenetic trees. *Journal of Molecular Evolution,* *25*, 351-360.

Glenn, T. C. (2011). Field guide to next-generation DNA sequencers. *Molecular Ecology Resources*, *11*, 759-769.

Hanslik, S., & Kruckenhauser, L. (2000). Microsatellite loci for two European sciurid species (*Marmota marmota, Spermophilus citellus*). *Molecular Ecology,* *9*, 2163-2165.

Klinkicht, M. (1993) Untersuchungen zum Paarungssystem des Alpenmurmeltiers, *Marmota M. marmota* mittels DAN Fingerprinting. Ph.D. thesis, University of Munich.

R Development Core Team (2013). R: A language and environment for statistical computing. R Foundation for Statistical Computing Vienna, Austria

Warnes, G. R. (2012). genetics: population genetics. R package version 1.3.8. http://CRAN.R-project.org/package=genetics
