# Supplementary material for: Mate choice for neutral and MHC genetic characteristics in Alpine marmots: different targets in different contexts?
Source: Ecol Evol. 2016 May 25;6(13):4243–57. doi: 10.1002/ece3.2189 (PMC4930977; doi:10.1002/ece3.2189)
Supplement: Supplementary file 2 — Appendix S2. Estimation of relatedness with pedigree and correlations between (1) the relatedness estimated with the pedigree and the relatedness estimated estimated with the Queller and Goodnight (1989) and (2) the relatedness estimated with the pedigree and the MHC proteic dissimilarity. [file ECE3-6-4243-s002.doc]

**Mate choice for neutral and MHC genetic characteristics in Alpine marmots: different targets in different contexts?**

**Appendix S2** Estimation of relatedness with pedigree and correlations between (1) the relatedness estimated with the pedigree and the relatedness estimated estimated with the Queller & Goodnight (1989) and (2) the relatedness estimated with the pedigree and the MHC proteic dissimilarity

*Methods*

To test whether neutral genetic compatibility estimated with the genetic characteristics at 16 microsatellites represent genome-wide patterns, relatedness was estimated based on pedigree information obtained with parentage analyisis. The pedigree-based relatedness was calculated using the function kin implemented in the R package “synbreed” (Wimmer *et al.,* 2012). Spearman's rank correlation coefficients were calculated between the pedigree-based relatedness and the Queller & Goodnight's relatedness. Also, to discard whether pedigree-based relatedness and MHC compatibility convey similar information, Spearman's rank correlation coefficients were calculated between the pedigree-based relatedness and MHC proteic dissimilarity.

*Results*

As expected, pedigree-based relatedness was correlated with Queller & Goodnight's relatedness (ρ = 0.45; CI 95% = [0.31, 0.56]; n = 158, P = <0.0001), suggesting that Queller & Goodnight's relatedness calculated at 16 microsatellite are representative of genome-wide patterns.

Pedigree-based relatedness and MHC compatibility estimators were not correlated regardless of the MHC loci considered (MHC class I proteic dissimilarity: ρ = -0.11; CI 95% = [-0.27, 0.05]; n = 148, P = 0.17; MHC class II proteic dissimilarity: ρ = 0.16; CI 95% = [-0.31, 0.01]; n = 152, P = 0.05), suggesting that MHC loci and pedigree-based relatedness convey rather different information.

**References**

Queller, D. C., & Goodnight, K. F. (1989). Estimating relatedness using genetic-markers. *Evolution*, *43*, 258-275.

Wimmer, V., Albrecht, T., Auinger, H. J., & Schön, C. C. (2012). synbreed: a framework for the analysis of genomic prediction data using R. *Bioinformatics*, *28*, 2086-2087.
