# Supplementary material for: Mate choice for neutral and MHC genetic characteristics in Alpine marmots: different targets in different contexts?
Source: Ecol Evol. 2016 May 25;6(13):4243–57. doi: 10.1002/ece3.2189 (PMC4930977; doi:10.1002/ece3.2189)
Supplement: Supplementary file 3 — Appendix S3. Effect of the spatial distance on males and pairs genetic characteristics. [file ECE3-6-4243-s003.doc]

**Mate choice for neutral and MHC genetic characteristics in Alpine marmots: different targets in different contexts?**

**Appendix S3** Effect of the spatial distance on males and pairs genetic characteristics

*Dispersal distance*

Since 1990, the dispersal distance of a total of 109 males and 94 females that reached dominance in the study site was known. A total of 83 (73%) out of these 109 males and 83 (88%) out of these 94 females came from the study site and have either inherited dominance in their natal territory, moved to an adjacent territory or to another territory inside the study site to reach dominance (Fig. S1), whereas the remaining 26 males (24%) and 11 females (12%) came from outside the study site (Fig. S1). The dispersal distance between the natal and the dominance territory was short, with a mean distance ± SD of 1.40 ± 1.01 for the 83 males and a mean distance of 0.95 ± 0.87 for the 83 females that were born and reached dominance inside our study site.

**Fig. S1** Dispersal distance (measured in the number of territories between the natal and the dominance territory) of males and females that reached dominance between 1990 and 2014 in the Nature Reserve of La Grande Sassière
